# Understanding the Genetic Architecture of Vitamin Status Biomarkers in the Genome-Wide Association Study Era: Biological Insights and Clinical Significance

**DOI:** 10.1016/j.advnut.2024.100344

**Published:** 2024-11-16

**Authors:** William R Reay, Erin D Clarke, Clara Albiñana, Liang-Dar Hwang

**Affiliations:** 1 Menzies Institute for Medical Research, University of Tasmania, Hobart, TAS, Australia; 2 Food and Nutrition Research Program, Hunter Medical Research Institute, New Lambton Heights, NSW, Australia; 3 School of Health Sciences, the University of Newcastle, University Drive, Callaghan, NSW, Australia; 4 Big Data Institute, University of Oxford, Headington, Oxford, United Kingdom; 5 National Centre for Register-based Research, Aarhus University, Aarhus, Denmark; 6 Institute for Molecular Bioscience, the University of Queensland, Brisbane, QLD, Australia

**Keywords:** Genome-wide association studies, vitamin status biomarkers, polygenic scores, Mendelian randomization, nutritional epidemiology

## Abstract

Vitamins play an intrinsic role in human health and are targets for clinical intervention through dietary or pharmacological approaches. Biomarkers of vitamin status are complex traits, measurable phenotypes that arise from an interplay between dietary and other environmental factors with a genetic component that is polygenic, meaning many genes are plausibly involved. Studying these genetic influences will improve our knowledge of fundamental vitamin biochemistry, refine estimates of the effects of vitamins on human health, and may in future prove clinically actionable. Here, we evaluate genetic studies of circulating and excreted biomarkers of vitamin status in the era of hypothesis-free genome-wide association studies (GWAS) that have provided unprecedented insights into the genetic architecture of these traits. We found that the most comprehensive and well-powered GWAS currently available were for circulating status biomarkers of vitamin A, C, D, and a subset of the B vitamins (B_9_ and B_12_). The biology implicated by GWAS of measured biomarkers of each vitamin is then discussed, both in terms of key genes and higher-order processes. Across all major vitamins, there were genetic signals revealed by GWAS that could be directly linked with known vitamin biochemistry. We also outline how genetic variants associated with vitamin status biomarkers have been already extensively used to estimate causal effects of vitamins on human health outcomes, which is particularly important given the large number of randomized control trials of vitamin related interventions with null findings. Finally, we discuss the current evidence for the clinical applicability of findings from vitamin GWAS, along with future directions for the field to maximize the utility of these data.

**Statement of Significance**

This is the first review to comprehensively evaluate the utility of hypothesis-free, genome-wide association studies to reveal genetic influences on vitamin status biomarkers across all major vitamins.

## Introduction

Vitamins are a group of organic, essential micronutrient compounds, meaning they are necessary in small quantities for a variety of biological functions and must be externally consumed. Biomarkers of vitamin status, that is, indices of whether an individual has physiological reserves within ranges considered nonpathological, are complex traits. We use the term “trait” forthwith in the same context it is used in the field of genetics, meaning a measured or observed phenotype. For some vitamins, these biomarkers represent directly measuring the abundance of the circulating or excreted compound, whereas others use proxies such as metabolites or enzymatic activity [[1], [2], [3]]. A proportion of the population variation in vitamin status is because of genetics (that is, heritability). This means that vitamin status arises because of an interplay between genetics and prominent dietary and nondietary influences. Understanding the different elements of the genetic architecture of vitamin status could accelerate the so-called “precision nutrition” [4]. However, progress in this area has previously been hampered by a reliance on “candidate gene,” hypothesis-driven studies that are greatly inflated for false positives [5], as well as small sample sizes and a lack of requisite statistical rigor. One exception of this has been in the field of high-impact, rare “monogenic” inborn errors in metabolism related to vitamins that are clinically actionable and have been reviewed elsewhere [[6], [7], [8], [9]].

**FIGURE 1:**
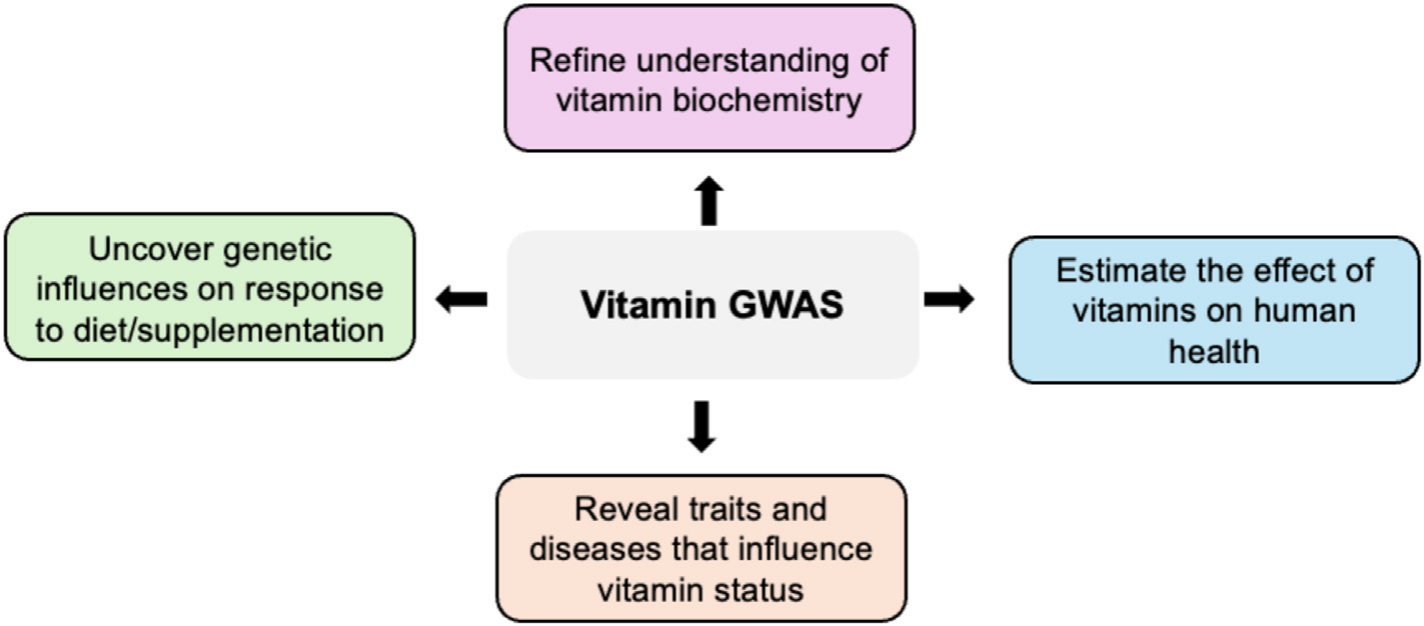
Examples of the utility of pursuing genome-wide association studies (GWAS) of biomarkers of vitamin status. GWAS of vitamin status may be both biologically and clinically informative for several reasons, four of which are briefly summarized in the above figure and discussed further throughout the review. GWAS, genome-wide association studies.

The current era of widespread, genome-wide association studies (GWAS), which are a hypothesis-free test of the association of millions of variants genome-wide with any trait of interest, can more reliably characterize specific genetic impacts on vitamins [10]. This is because GWAS can prioritize the most confident signals after appropriate multiple-testing correction, as well as leverage the “polygenic” signal across the genome to provide biologically meaningful insights at both the population and individual levels (Figure 1). Genetic propensity for sufficient vitamin status can be indexed in an individual using a polygenic scoring approach that summates the estimated effect of alleles on vitamin biochemistry across the genome [11]. The utility of these scores will vary, with the heritability estimated from GWAS (often referred to as single nucleotide polymorphism (SNP) heritability) as the theoretical upper bound of their prediction accuracy [12]. Genetic variants associated with the concentration of vitamin status biomarkers can also have important epidemiological utility, particularly as large randomized control trials (RCTs) [13] have by and large failed to demonstrate the value of vitamin supplementation for individuals not deficient. For example, Mendelian randomization (MR) is a popular technique in genetic epidemiology that leverages genetic variants associated with an exposure variable, such as the abundance of a vitamin status biomarker, to infer causal exposure–outcome relationships [[14], [15], [16], [17]]. Specifically, vitamin-associated variants can be leveraged as instrumental variables (IVs) that proxy the vitamin, and because of Mendel’s laws of independent assortment and random segregation, these genetic IVs can be used to estimate causal relationships, provided a series of assumptions regarding IV validity are met [15,16]. While the application of MR can be statistically challenging in the presence of pleiotropy and other confounders, it has shown great potential to enhance the selection of suitable candidates for RCT, as well as minimize unnecessary trials [18,19]. For instance, MR studies of high-density lipoprotein predicted a lack of benefit in raising HDL pharmacologically for coronary artery disease shown by several large-scale RCT, whereas observational studies consistently indicated the opposite [20]. These principles of genetically informed causal inference can also be utilized to better understand traits and disorders that influence the homeostasis and biochemistry of vitamins. In this review, we will discuss the progress to date that GWAS has made in characterizing the genetic architecture of biomarkers of vitamin status. The biological significance of these findings will be evaluated, as well as any evidence of current or future clinical utility.

### Available GWAS of vitamin status biomarkers

#### Vitamin A

Vitamin A is fat soluble and refers to a group of compounds that are chemically related. Retinol (all-*trans* retinol) and retinyl ester represent the primary dietary sources of vitamin A from animal products [21]. Plant-based materials also are sources of vitamin A through carotenoid compounds that are reversibly converted to retinaldehyde, an oxidized metabolite of retinol [22,23]. However, only carotenoids with ≥1 unsubstituted β-ionone ring are able to be converted to retinaldehyde via an irreversible oxidative cleavage reaction [24,25]. The dietary sources, physiology, biochemistry, and clinical significance of vitamin A family compounds have been reviewed extensively elsewhere [21,26]. The exploration of vitamin A genetics is supported by the pharmacological actionability of retinoid biology in certain indications, along with the paucity of evidence regarding the clinical significance of vitamin A concentrations within normal established ranges. Circulating plasma or serum retinol has some utility as a biomarker of vitamin A status (more so at the population level), despite limitations because of tight homeostatic control and impacts of factors such as inflammation and protein malnutrition [27]. Circulating retinol, along with carotenoids, have been subjected to GWAS to characterize the genetic architecture of these traits, as discussed in the proceeding section.

#### Current findings from GWAS of circulating vitamin A compounds

**FIGURE 2:**
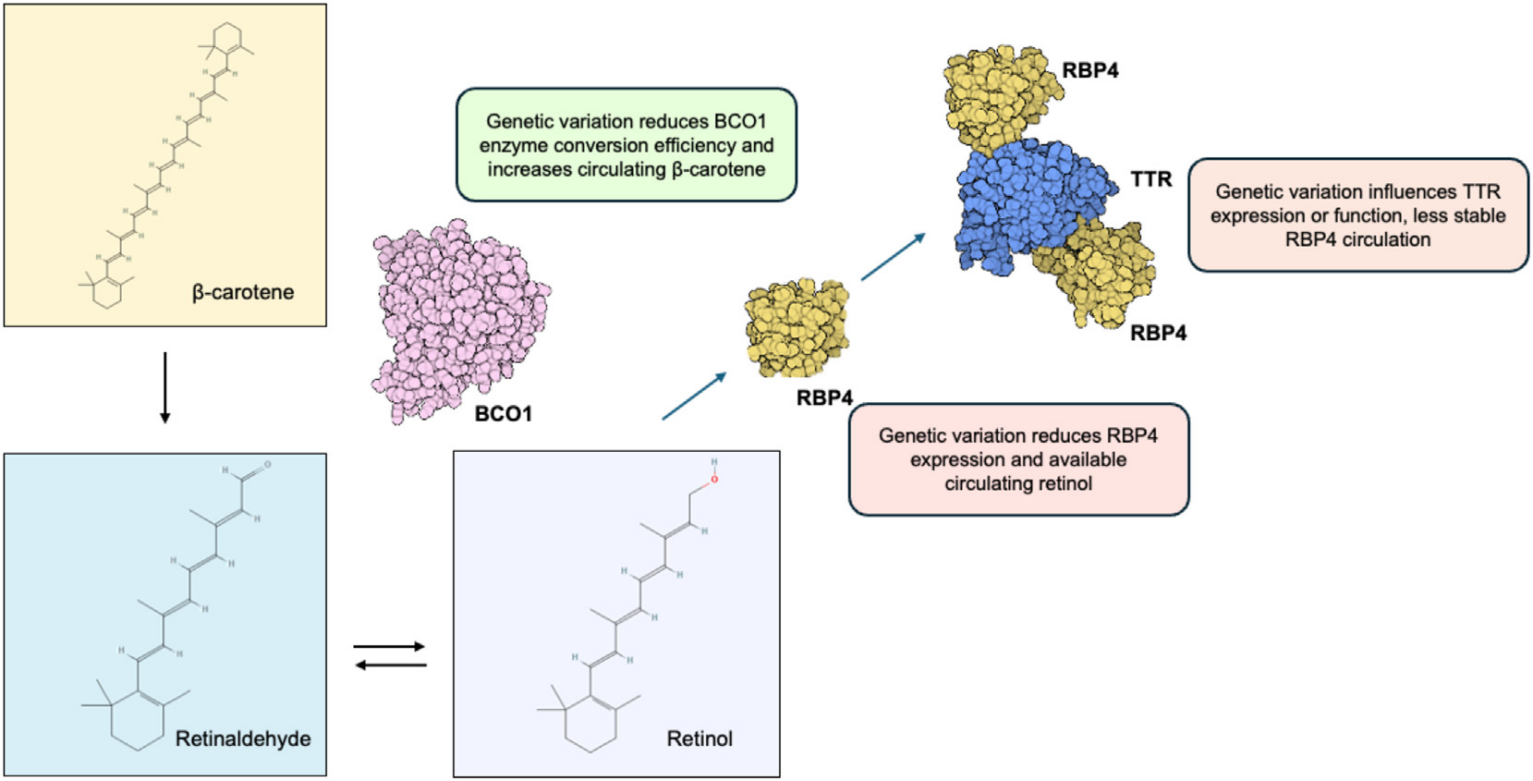
Putative mechanism of key common variants associated with vitamin A compounds from GWAS. Provitamin A carotenoids such as β-carotene can be cleaved into two retinaldehyde (two molecules) in a reaction catalyzed by the enzyme Beta-Carotene 15,15′-monooxygenase 1 encoded by BCO1. Variation physically mapped to BCO1 associated with circulating β-carotene at genome-wide significance has been suggested experimentally to reduce the efficiency of enzymatic conversion to retinaldehyde [32]. As a result, this signal is associated with a small-to-moderate increase in circulating β-carotene. Common variation in RBP4, which encodes the primary circulating transporter of retinol, retinol binding protein 4, is associated with circulating retinol at genome-wide significance. The same causal variant has been estimated to underly both circulating RBP4 expression and circulating retinol (colocalization) [28], suggesting that the allele which decreases RBP4 abundance in turn decreases retinol transport from the liver. RBP4 complexes with transthyretin (encoded by TTR) to stabilize the complex and prevent excessive renal filtration of RBP4. Variation mapped to TTR has also been associated with circulating retinol, and while the effect of these variants on expression or function of TTR has not been fully resolved, it is logical that retinol decreasing alleles would impact TTR abundance or function, therefore, resulting in increased renal filtration of RBP4 bound retinol. Chemical formulas sourced from PubChem (https://pubchem.ncbi.nlm.nih.gov/). GWAS, genome-wide association studies.

The largest GWAS to date of circulating retinol was recently published, with the sample size exceeding 20,000 for the first time for a vitamin A compound [28]. This study identified 8 genome-wide significant common frequency signals associated with circulating retinol, 6 of which being novel, as well as a rare frequency signal. The largest previous dedicated GWAS of circulating retinol from Mondul et al. [29] (*N* ∼ 5000) had already implicated 2 genes, as defined by the nearest gene to the lead variant, retinol binding protein 4 (*RBP4*) and transthyretin (*TTR*). These 2 genes have a clear and unambiguous relationship with circulating retinol as they form the primary transport complex for retinol in serum (Figure 2)[28,[30], [31], [32]]. The recent Reay et al. [28] GWAS further strengthened the confidence of the association signal mapped to these genes, with reported additive effect sizes for each signal of ∼ |0.1| standard deviations (SDs) in retinol per allele. While these observed effect sizes are small relative to factors such as sex, age, and comorbid medical conditions, they are somewhat large relative to common frequency GWAS signals for many other complex traits, particularly binary phenotypes. Probabilistic fine-mapping and functional annotation suggested noncoding regulatory perturbations likely confer the effect of these variants. The remaining 6 loci implicated by the latest GWAS were also plausibly mapped to relevant biology, such as a known hotspot for association with metabolic traits in the gene encoding glucokinase regulatory protein (*GCKR*) that influences the pleiotropic role of glucokinase in lipid and glycemic biology, among others. The pathway analysis of genes prioritized at each of the common variant GWAS signals, as well as high-confidence signals from gene-based approaches to boost power, supported that common variant influences on circulating retinol were enriched with processes related to the regulation of carbohydrate metabolism (for example, genes such as *GCKR*, *MLXIPL*, and *GSK3B*), although these genes are all known to be related to hepatic energy metabolism more broadly. Indeed, the salience of the liver for genetic effects on circulating retinol is also supported by a strong upregulation of the expression of prioritized associated genes in the liver. The rare variant signal uncovered in this GWAS was less biologically interpretable (an intergenic variant with its closest transcription start site *COX7C*, which encodes a mitochondrial respiratory chain subunit). As its effect size was somewhat larger than any common variant lead SNP, with an estimated ∼0.4 SD reduction in circulating retinol per minor allele, it warrants further investigation. Overall, genome-wide SNP heritability estimated from summary statistics was still noisy for circulating retinol at current sample sizes, ranging between 7% and 15% depending on the modeling parameters used, although heritability of retinol directly estimated from individual genotypes of a small American study (*N* ∼ 1700) was almost 30% [33]. A polygenic score (PGS) of circulating retinol generated by the latest GWAS explained a small (∼2%) but statistically significant proportion of the variance in circulating retinol using an independent sample, suggesting that further work is needed to increase sample size and maximize the utility of genetically predicted retinol for studying phenomenon such as gene-by-environment interactions (GxE). Common variant signals associated with circulating retinol have also been used to estimate causal relationships with a diverse range of phenotypic outcomes using MR. The most comprehensive such study was a hypothesis-free, phenome-wide MR study included in the Reay et al*.* GWAS [28]. Importantly, this genetics-informed approach was able to prioritize known beneficial and adverse clinical effects of vitamin A signaling, including on the eye, the immune system, and the heart [[34], [35], [36]]. Genetically predicted causal effects of retinol were also found on other important endpoints of potential clinical interest, including adult brain structure and the microbiome, the latter of which is subject to an increasing number of intervention studies with respect to vitamin A supplementation, as reviewed elsewhere [37]. Reverse effects on circulating retinol as the outcome were also examined, supporting the known interrelationship between lipids and retinoid metabolism [38], as well as a retinol increasing effect of serum creatinine that could be mediated by reduced kidney function or nonrenal factors related to muscle/fat mass.

GWAS have also been performed on circulating provitamin A carotenoids that can be cleaved into retinaldehyde, albeit with much smaller sample sizes and less specific interrogation of the findings. In 2009, Ferrucci et al. [39] published a GWAS of circulating carotenoids in a discovery cohort consisting of ∼1190 individuals, followed by replication in ∼2800 individuals. The strongest signal uncovered in that study was a genome-wide significant signal associated with circulating abundance of β-carotene that is located upstream of the gene Beta-Carotene 15,15'-Monooxygenase 1 (*BCO1*), with the rs6564851 lead SNP estimated to explain almost 2% of the variance in circulating β-carotene, which is a nontrivial effect for a common frequency locus. *BCO1* is clearly a plausible gene that influences β-carotene as it is the enzyme that catalyzes oxidative carotenoid cleavage into retinaldehyde (Figure 2) [40]. This signal has also been investigated in subsequent studies to mechanistically interpret this effect, with evidence that conversion efficiency of the enzyme is decreased, and thus, results in an elevated abundance of β-carotene that is not cleaved into a retinoid [30,41]. Interestingly, the lead SNP is in relatively high linkage disequilibrium (LD) in Europeans (LD *r*^2^ ≈ 0.7) with a missense variant rs6420424 in *BCO1* that may explain the effect on enzymatic conversion efficiency, although further investigation is needed. The rs6564851 lead SNP was also nominally associated with increased α-carotene, but not β-cryptoxanthin or retinol [39]. An absence of an effect of this *BCO1* allele on circulating retinol is plausibly because of the buffering effects of hepatically stored retinyl ester, as well as preformed retinol likely consumed through dietary animal products. Recent high-throughput metabolomics studies have provided some further genetic data on the provitamin A carotenoid β-cryptoxanthin. However, dedicated analyses such as that applied to retinol are still lacking and sample sizes are still small (*N* < 10,000), with no genome-wide significant signals found by those studies [[42], [43], [44]]. Sample sizes for carotenoids are also still insufficient for summary statistics-based heritability estimates.

#### Potential clinical implications and future directions from vitamin A GWAS

The most confidently estimated component of genetic influences on circulating markers related to vitamin A status is undoubtedly the relationship between the RBP4:TTR complex and circulating retinol, for which an independent signal exists within each component. Given the homeostatic buffering of retinol from hepatic sources, the effect sizes of variants in this context may not be directly clinically informative for individuals. However, individuals homozygous for both retinol decreasing signals in *RBP4* and *TTR*, respectively, would be predicted to carry 0.4 SD less circulating retinol under an additive model. While RBP4:TTR double homozygotes would be rare, these individuals may warrant further consideration if they are a member of a population at increased risk of vitamin A deficiency. Retinol PGS may additionally be useful in future for this purpose and index a larger effect for a greater percentage of the population with decreased genetically predicted retinol but may be less biologically specific than just signals in the RBP4:TTR complex. As serum retinol is often only poorly correlated with dietary intake because of hepatic stores, more work is also needed to characterize how these variants relate to overall retinol status and if there is any interplay with diet. One finding from retinol GWAS that is of potential clinical interest are the putative causal relationships uncovered by MR. One such example are the MR causal estimates of genetically predicted circulating retinol on the microbiome. Emerging data from recent microbiome related clinical trials of vitamin A supplementation should be integrated with specific relationships identified by genetically informed causal inference to further refine potential positive or adverse effects of vitamin A supplementation or vitamin A rich dietary patterns on the microbiome [37]. In terms of provitamin A compounds, the *BCO1* beta-carotene signal remains the only tangible aspect of their genetic architecture at present. Previous small-scale studies suggest that carriers of *BCOM1* nonsynonymous variants may exhibit impaired conversion of beta-carotene to retinol, as indexed through reduced retinyl palmitate [45]. In the Ferrucci et al. [39] carotenoid GWAS, the *BCOM1* signal was not associated with circulating retinol, likely because of buffering from hepatic stores. However, for individuals who follow a dietary pattern without the consumption of preformed vitamin A from animal products, the effect of this reduction in *BCO1* beta-carotene cleavage efficiency on retinol status may be more relevant and warrants further investigation. Overall, larger samples are still required to fully realize the genetic architecture of vitamin A, particularly as SNP heritability estimates for retinol remain statistically noisy in the current largest GWAS of serum retinol measurement [28]. However, increased sample size is not the only area in the field of vitamin A genetics that requires additional effort given the limitations of circulating retinol in serum as a biomarker of retinol status. Ideally, future studies should attempt to capture genetic effects on tissue-specific measures [for example, skin, urine, and cerebrospinal fluid (CSF)], relative dose response tests that assess adequacy of liver stores, and abundance of key retinol metabolites such as all-*trans* retinoic acid in tissues of particular clinical interest, such as the central nervous system. Additional rare variant data and effects of structural variants and tandem repeats would also greatly enhance our understanding of vitamin A genetic architecture, with evidence of high-impact “monogenic” impacts on retinoid biology through genes such as the cellular retinol receptor gene *STRA6* [46].

### B Vitamins

The B vitamins encompass a class of 8 water-soluble vitamins that while often consumed from similar dietary sources, are diverse in their chemical structure [47,48]. B vitamins (B_1_, B_2_, B_3_, B_5_, B_6_, B_7_, B_9_, and B_12_) are also grouped together in this fashion because of their activity as intracellular coenzymes/cofactors, or as precursors thereof. We do not extensively outline the biochemistry, synthesis, and function here of all 8 B vitamins because of space constraints; however, this has been described extensively elsewhere [47,49,50]. By way of an example, vitamin B_12_ (cobalamin) acts as a cofactor for the enzymes methionine synthase and methylmalonyl-CoA mutase, both of which have essential functionality in cellular metabolism [51,52]. The assessment of intake and status of B vitamins can be completed through dietary assessment questionnaires, such as 24-h recalls, food records, or food frequency questionnaires, linked to a relevant food and nutrient database with region-specific nutrients. Additionally, biological markers are used to assess status. However, the lack of informative biomarkers for some B vitamins has hindered the progress in unraveling their genetic architecture, as outlined below.

#### Current findings from vitamin B_1_ (thiamine) GWAS

There has been at the time of writing still very limited genetic investigation of vitamin B_1_ (thiamine) status biomarkers using GWAS. Studying this vitamin is further complicated by the inadequacy of serum or urine thiamine as a biomarker of thiamine status, or its phosphorylated active forms, as they only reflect recent intake [53]. Thiamine has nonetheless been measured in some small sample size high-throughput metabolomics GWAS such as that derived from urine by Schlosser et al. [44] (*N* ∼ 4200), although no genome-wide significant signals were found. Thiamine diphosphate saturation of the thiamine-dependent enzyme transketolase in erythrocytes is considered a biomarker of status [54], although subjecting this phenotype to GWAS will be difficult at sufficient sample sizes.

#### Current findings from vitamin B_2_ (riboflavin) GWAS

The vast majority of vitamin B_2_ (riboflavin) in the body exists as the coenzymes flavin mononucleotide (FMN) and flavin adenine dinucleotide (FAD) [55]. Analogous to vitamin B_1_, plasma and urine FAD/FMN only reflects recent intake as there is very limited storage. This has limited the application of GWAS. Riboflavin deficiency is often measured using the erythrocyte glutathione reductase activity coefficient that is indicative of riboflavin tissue saturation [56], although no known GWAS of this measure has been performed at the time of writing.

#### Current findings from vitamin B_3_ (niacin) GWAS

Vitamin B_3_ encompasses a family of vitamers that are all commonly referred to using the umbrella term of niacin. These vitamers (niacin, nicotinamide, and nicotinamide riboside) are all able to be converted into the coenzyme nicotinamide adenine dinucleotide that plays an intrinsic role in cellular metabolism [57]. A biochemical assay of vitamin B_3_ status is considered most effective through measurement of major niacin metabolites [1-methylnicotinamide (1-MN) and 1-methyl-2-pyridone-5-carboxamide (2-PYR)] that are excreted in urine (ideally a 24 h urine sample), with plasma concentrations of these metabolites not conclusively shown to be useful [3]. Several high-throughput plasma or urine metabolomics studies have subjected 1-MN and 2-PYR to GWAS, although all studies have had sample sizes of <10,000 and genome-wide significant loci have only been found for blood derived measurements. However, some of signals identified do present plausible links to niacin biochemistry—for example, the rs17322446 lead intronic lead SNP in *ACMSD* is associated with both decreased plasma 1-MN and 2-PYR in the Finnish METSIM GWAS [43]. *ACMSD* encodes the enzyme aminocarboxymuconate-semialdehyde decarboxylase that is known to play a role in the synthesis quinolinic acid, a precursor to niacin [58]. This *ACMSD* signal is also associated with several clinically relevant phenotypes, including hypertension and lung function, although the mediating role of niacin metabolites, ideally using SNP effect size estimates from urine samples, requires further investigation through approaches such as MR and probabilistic colocalization.

#### Current findings from vitamin B_5_ (pantothenic acid) GWAS

Given the ubiquitous nature of vitamin B_5_ (pantothenic acid), deficiency or toxicity has not been thoroughly studied and its health implications are not well understood, although there is some suggestive evidence from small-scale RCTs of triglyceride lowering effect of high-dose pantethine, a pantothenic acid metabolite [59]. Vitamin B_5_ is mostly found complexed with coenzyme A, and therefore, enzyme catalyzed liberation from these complexes in leukocytes or erythrocytes is a potential biomarker but evidence is still lacking [2]. As there remains a lack of consensus as which biomarkers of vitamin B_5_ are clinically meaningful, GWAS of markers of B_5_ status will not be particularly informative until these matters are further resolved.

#### Current findings from vitamin B_6_ GWAS

Vitamin B_6_ is an umbrella term for a group of 6 vitamers classified as having B_6_ activity. The active form, pyridoxal-5′-phosphate (PLP), acts as a coenzyme in a diverse range of enzymatic reactions [60], with plasma/serum PLP considered a somewhat informative marker of status [61]. An early circulating vitamin B_6_ GWAS with a discovery cohort sample size of ∼1200 participants found 1 genome-wide significant locus on chromosome 1 containing the alkaline phosphatase (*ALPL*) gene [62]. This is a directly biologically interpretable signal as B_6_ transport across the cell membrane is preceded by hydrolysis of B_6_ vitamers by membrane-bound ALPL [63]. In a small follow-up study (*N* = 493) that leveraged both plasma and CSF measurements, homozygotes for the *ALPL* lead SNP displayed ≤1.6 times higher ratio (CSF) between PLP and pyridoxal (PL), suggesting reduced hydrolysis of PL to PLP by ALPL, likely resulting from reduced ALPL activity or expression [64]. A larger study in 2019, albeit still small (*N* ∼ 2232), further supported the *ALPL* signal, and found an additional signal for PLP:PL that includes a missense variant in the gene pyridine nucleotide disulfide oxidoreductase domain 2 (*PYROXD2*), although the function of this signal remains poorly characterized [65]. Overall, further work is warranted to investigate whether signals in *ALPL* are moderated by dietary intake in terms of their association with PLP:PL. Unlike the previously discussed B vitamins, vitamin B_6_-associated variants have been used as instruments for causal inference in some quite comprehensive previous MR studies. In a UK Biobank (UKBB) phenome-wide MR using the *ALPL* IV, there was some evidence of a protective effect of genetically predicted vitamin B_6_ on the odds of calculus of the kidney and ureter that was nominally replicated using an independent outcome GWAS from FinnGen [66]. This finding does have some previous supporting evidence [67]; however, sensitivity analyses testing colocalization (same underlying causal variant/s) of the *ALPL* locus are warranted to ensure that this association does not arise because of an indirect association through linkage [17]. Further, the *ALPL* IV will plausibly also influence other biochemistry unrelated directly to B_6_ vitamers, and therefore, larger samples are needed to increase the sensitivity and specificity of MR approaches. Several other studies have also used the *ALPL* IV to investigate causal relationships, such as with the odds of cancer, sepsis, and cardiovascular disease [[68], [69], [70]]. Findings from the application of these models were largely null, except for some weak nominal evidence for a protective effect of B_6_ on stroke, from which definitive conclusions cannot be drawn.

#### Current findings from vitamin B_7_ (biotin) GWAS

There still remains a paucity of evidence to support the generalized application of biomarkers indicative of vitamin B_7_ (biotin) status [71]. Naturally, there is also an absence of dedicated GWAS. A 24-h urinary excretion of the compound 3-hydroxyisovaleric acid (3-HIA) has some evidence for utility as a biomarker of biotin status as it is an indirect byproduct of insufficient biotin available for carboxylase mediated leucine catabolism [71,72]. While no GWAS were available of 3-HIA in urine at the time of writing, recent high-throughput GS-MS profiling of plasma samples from a Japanese cohort has provided a GWAS of the abundance of 3-HIA in blood [73]. While the implications of plasma 3-HIA as a biotin biomarker are less clear, three genome-wide significant signals were uncovered. Overall, we still have no clear indicators of the heritability of genetic architecture of biotin abundance and status.

#### Current findings from vitamin B_9_ (folate) GWAS

Vitamin B_9_ (folate) collectively refers to several forms of folic acid and related compounds, with the primary active form being tetrahydrofolic acid. While plasma/serum folate is commonly used in laboratory testing of folate status, it is sensitive to recent intake; as a result, erythrocyte-derived folate measurements provide a more stable marker of status[74]. Folate biology has been the subject of significant interest in terms of genetic influences before the era of widespread adoption of GWAS. Specifically, the well-known 5,10-methylenetetrahydrofolate reductase (*MTHFR*)-C677T (aka c.665C → T or the “thermolabile” variant) common polymorphism (rs1801133) was implicated before the GWAS era to influence folate status [75,76]. Mechanistically, MTHFR catalyzes the conversion of 5,10-methylenetetrahydrofolate to the primary circulating folate form 5-methyltetrahydrofolate, with rs1801133 shown in vitro to influence enzymatic activity. This variant and other *MTHFR* genotypes have been studied extensively in many hypothesis-driven follow-up studies in terms of its clinical actionability and relationship with disease outcomes. However, while the biochemical mechanism of *MTHFR* variation on folate biology is clear, the clinical actionability of this has not been established and remains an ongoing controversy. This is particularly pertinent given the importance of folate in the prevention of neural tube defects [77]. For example, a 2013 American College of Medical Genetics (ACMG) practice resource report concluded that there was a lack of strong evidence to support routine testing for *MTHFR* variation such as the “thermolabile” variant [78], a position also supported by the American College of Obstetricians and Gynaecologists in terms of adverse pregnancy outcomes [79]. The ACMG publication does state that pregnant women who are already known to be homozygotes for the “thermolabile” variant could be counseled relative to a modestly increased risk of an offspring with neural tube defects, although, crucially, there is no strong evidence that dietary or supplementation related interventions are warranted beyond what is recommended regardless of genotype in this context. The European Food Safety Authority in scientific opinions published in 2014 and 2023 do suggest that there is some emerging evidence to suggest the need for higher folate requirement of “thermolabile” *MTHFR* homozygotes [80], mainly citing a randomized control (*N* ∼ 932) which suggested that “thermolabile” *MTHFR* homozygotes did not achieve the same erythrocyte folate measurements after supplementation [81]. The authors of this review do not make any specific recommendations beyond what has been established without new evidence to suggest the direct clinical actionability of *MTHFR* variation and the need for population screening, which has not been shown thus far.

In terms of folate GWAS, the largest known study of erythrocyte folate, the more informative measure, comprised of ∼2200 Irish participants and revealed the same *MTHFR* variant as the only genome-wide significant signal [82]. The largest serum folate GWAS to date was drawn from deep-sequenced Scandinavian populations in ∼37,000 participants that uncovered 2 genome-wide significant loci [83]. In an Icelandic subset of that study comprising sibling pairs, heritability of serum folate was estimated in the region of 17%. Unsurprisingly, the “thermolabile” *MTHFR* variant was the strongest signal with an additive estimated effect size of ∼ |0.1| SD (Icelandic subset) to ∼ |0.18| SD (Danish subset) on serum folate, which interestingly is not markedly larger than many other vitamin-associated loci relative to the significant attention given to this locus in prior studies, although there is a previous suggestion of nonadditivity of this locus’ effect. The second folate associated signal was plausibly mapped to the gene that encodes folate receptor gamma (*FOLR3*), which mediates the transport of 5-methyltetrahydrofolate into the cell. This study also conducted a phenome-wide association study using endpoints in the deCODE Icelandic resource and found suggestive evidence of an association between the “thermolabile” *MTHFR* variant and thoracic aortic aneurysm using a recessive model, although this did not survive multiple-testing correction. The *MTHFR* and *FOLR3* signals have been used as genetic instruments in a phenome-wide study of genetically predicted circulating folate on UKBB outcomes [66]. While a PGS of serum folate constructed from the two signals was associated with cardiovascular endpoints, including a protective effect on the odds of hypertension and hypercholesterolemia, this was not statistically significant using an MR approach using an independent non-UKBB GWAS. This discrepancy could arise from the MR approach also weighting the precision (standard error) of the SNP effects on folate in the calculation of the test statistics as opposed to the PGS weighted only by effect size. In a more directed MR study of autoimmune disease, there was some evidence of a protective effect of serum folate on the odds of vitiligo that was not driven by either the *MTHFR* or *FOLR3* variant alone [84]. Notably, another study also found some weak evidence for a protective effect of folate on cardiovascular disease, although this did not survive multiple-testing correction [70]. Larger sample size folate GWAS would assist in refining more putative genetic instruments that could be used in causal inference, particularly as with only two genome-wide significant instruments currently available, the application of several MR methods is not feasible.

#### Current findings from vitamin B_12_ (cobalamin) GWAS

Vitamin B_12_ (cobalamin) structurally contains a corrin ring with a cobalt atom center, hence the term cobalamin. An interesting facet of vitamin B_12_ biology is that it can only be found naturally from animal products; however, individuals can also maintain status through supplementation or fortified foods. Serum cobalamin is a widely used biomarker of long-term status, although other related biochemical markers can be used in combination for more specific insights and there are some questions regarding the sensitivity of serum cobalamin [1,85]. The largest GWAS of circulating vitamin B_12_ concentration is drawn from the same Scandinavian cohorts as the folate study described above [83]. Here, the heritability of B_12_ (∼27%) was higher than that of folate, with 11 genome-wide significant loci associated with this vitamin. A common missense variant in *FUT2* was the most statistically significant finding, with each A allele of rs602662 (missense lead SNP) associated with ∼0.2 SD increase in B_12_. This signal is related to ABO secretor status, which refers to the presence of Lewis ABO blood group antigens on epithelial surfaces and in bodily fluids. ABO secretor status has some purported clinical significance as “non-secretors” exhibit reduced risk of norovirus infection, the lead cause of gastroenteritis [86]. The lead SNP of this signal is in relatively strong LD with the *FUT2* nonsense SNP W143X (rs601338)—likely the causal variant for its impact on B_12_ concentrations that is quite common (>30%) in all global ancestral superpopulations with the exception of East Asian ancestry populations for which a different “secretor” allele in *FUT2* occurs at a similar common frequency (rs1047781) [87]. One proposed explanation for the relationship between secretor status and circulating vitamin B_12_ relates to infection with *H*. *pylori* and its impact on vitamin B_12_ absorption that is modulated by this signal [87]; however, this was challenged by later work that suggested that haptocorrin bound B_12_ (only hepatic uptake) is associated with the *FUT2* secretor allele but transcobalamin bound B_12_ (bioactive form taken by all tissues) was not [88]. In that study, Velkova et al. [88] instead propose that this effect on haptocorrin bound B_12_ is because of an impact on hepatic uptake arising from altered glycosylation of haptocorrin bound B_12_. The aforementioned study of the bioactivate transcobalamin bound B12 (holo-transcobalamin) also found signals in genes uncovered by the largest Grarup et al. [83] GWAS of serum B_12_ (*CD320*, *TCN2*, *CUBN,* and *MMUT*), suggesting at least some consistency in genetic factors that influence the active form. The Grarup et al. [83] GWAS of circulating B_12_ concentrations also uncovered a rare missense variant in *MMACHC* (methylmalonic aciduria and homocystinuria type C protein) involved in vitamin B_12_ uptake that is associated with a relatively large effect (∼0.5 SD per allele). Interestingly, of the 11 reported loci in that GWAS, 8 of these loci (based on the closest gene) are members of the Reactome pathway *Cobalamin (Vitamin B*_*12*_*) transport and metabolism* (*CD320*, *TCN2*, *ABCD4*, *MMAA*, *MMACHC*, *TCN1*, *CUBN*, and *MMUT*). This demonstrates most of the significant genetic signals for B_12_, even at relatively limited sample sizes, have a clear link to the biochemistry of this vitamin, a finding not seen for several other micronutrients. GWAS have also been performed on other relevant circulating measures relevant to B_12_, such as methylmalonic acid (MMA), although the strongest genetic signals for MMA are somewhat distinct from circulating B_12_ [89].

The availability of several genome-wide significant variants with direct links to B_12_ biology renders B_12_ a good candidate for causal inference using MR. Two phenome-wide MR studies of circulating vitamin B_12_ using UKBB outcomes have been published [66,90]. A notable difference between these 2 studies is that Dib et al. [90] excluded the *FUT2* and *FUT6* vitamin B_12_ associated alleles as IVs because of their pleiotropic nature, which likely renders estimates from that study more interpretable. In Dib et al. [90], a phenome-wide scan of the UKBB using a B_12_ PGS was firstly conducted that found a somewhat informative relationship with the outcome “Vitamin B-complex deficiencies” (OR ∼ 0.73 per SD in score) despite the low power of that phenotype given it was defined using ICD-10 codes, an underestimate of the true burden of deficiency in the UKBB. This effect size was validated using MR where the effect size was larger, as well as a logical relationship with megaloblastic and pernicious anemia [91]. While this phenome-wide approach is well controlled for type 1 error, the limitations of the ICD-10 derived phenotypes in the UKBB warrants this approach to be applied in other similar biobanks, particularly such as FinnGen, where Firth regression is used to better account for case/control imbalances and boost discovery power in this scenario.

#### Potential clinical implications and future directions from B vitamin GWAS

*MTHFR*-related variation and its impact on folate biology have received by far the most amount of attention with respect to genetic influences on B vitamin status—however, there is no current evidence to support the utility of routine testing for *MTHFR* variants or *MTHFR* genotype-guided changes in clinical practice. With larger samples, a folate PGS may provide insights that exhibit a larger effect size than individual *MTHFR* variants which conceivably could be relevant to consider for indications such as pregnant women at increased risk of having offspring with neural tube defects, although evidence to directly support this is still lacking. Genetically informed causal inference using genetic proxies associated with the concentration of B vitamin status markers will be useful to further resolve the clinical utility of supplementation and, crucially, strengthen evidence to fail to reject the null hypothesis when RCTs are null. From a clinical dietetics perspective, there is also little research that has explored the benefits of dietary sources of B-vitamins—with most intervention studies focusing on supplementation or fortification [92,93]. Genetics could play a role in this future work in this area as supplemental forms of some B vitamins are metabolized differently to those found in dietary sources, and genetic influences may load differently onto aspects of metabolism more related to dietary intake compared with supplementation. Overall, it remains unclear whether response to B-vitamin dietary intake is modulated by genetics, as indexed by vitamin B status biomarkers, and whether there are direct clinical implications. For instance, the common variant signal in *ALPL* is associated with B_6_ vitamers, with in vivo animal data suggesting that diet can influence *ALPL* expression and high penetrance *ALPL* alleles linked in case reports to B_6_-responsive seizures in humans [94,95]. We conclude that the vitamin B_12_ represents the B-vitamin with the most potential at present for revealing biologically and clinically tangible insights from its genetic architecture. B_12_ PGS appear to be a good candidate for further investigation, given the interpretable biology of most of the identified B_12_-associated signals. One potential use scenario relates to B_12_ deficiency arising from plant-based diets and/or malabsorptive conditions. While a B_12_ PGS has not been investigated yet in this context, in Tanwar et al. [96], carriers of the B_12_ associated rs602662-G allele in *FUT2* appeared to have a larger point estimate of B_12_ lowering in participants who followed a vegetarian diet. However, we caution that this was a small sample-size study that did not explicitly test for a nonadditive GxE effect using best statistical practices [97], and replication is warranted, ideally using a B_12_ PGS as the genetic variable. An increase in sample size for all B vitamins with interpretable biomarker measures would be useful to further genetic investigation of these traits, with particular attention paid to boosting diversity from genetically defined ancestral groups. For some B vitamins (B_1_, B_2_, B_5_, and B_7_), genetic studies will not be interpretable without further work refining the most appropriate status biomarkers that could be assayed in large enough sample sizes for GWAS.

### Vitamin C

Vitamin C (L-enantiomer of ascorbic acid) is a water-soluble vitamin that cannot be synthesized endogenously by humans, and therefore, must be obtained exogenously from diet [98]. A detailed review of the intake, biology, and clinical significance of vitamin C can be found elsewhere [99]. Plasma vitamin C is routinely used as a biomarker of vitamin C status, as well as a biomarker of intake of fruits and vegetables as these are the main sources of vitamin C in the diet [100]. The existence of a plasma biomarker has enabled GWAS of vitamin C in cohorts sufficiently large to discover genome-wide significant loci.

#### Current findings from GWAS of circulating vitamin C

**FIGURE 3:**
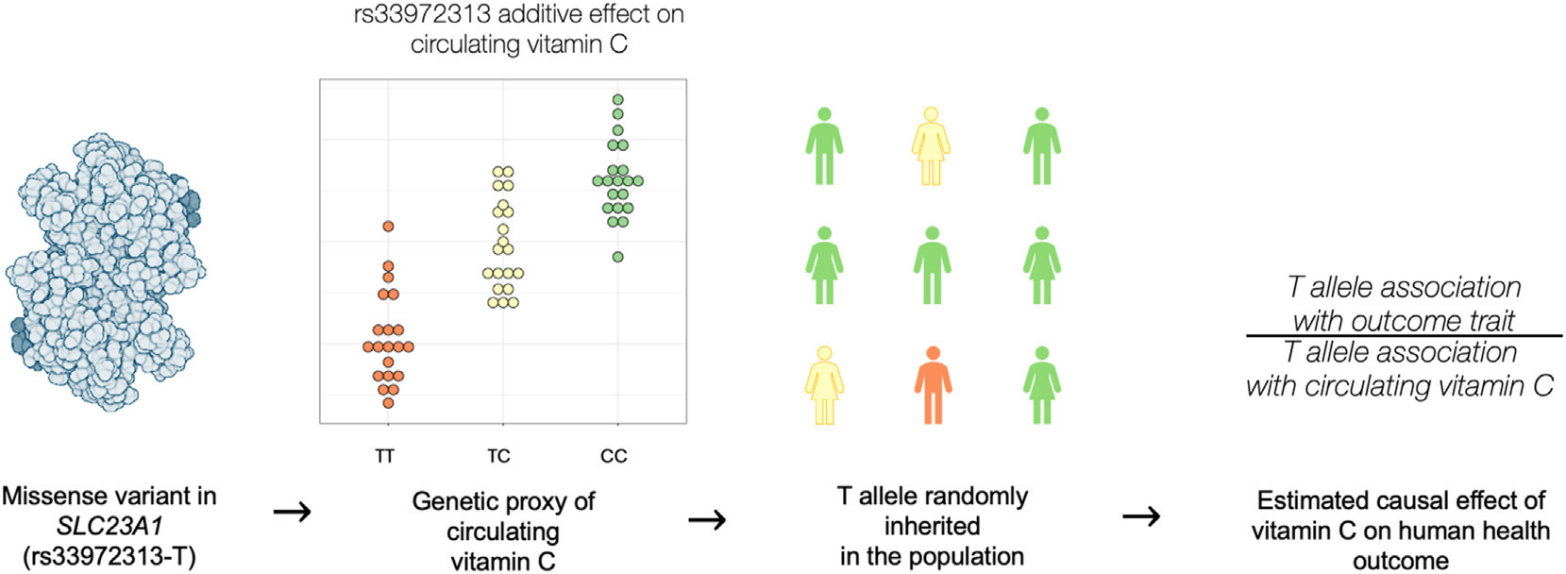
Schematic of how genetic variants associated with circulating vitamin C can be used for causal inference via Mendelian randomization. The rs33972313 missense variant in the transporter SLC23A1 is directly mechanistically linked to circulating vitamin C, whereby each T allele is associated with reduced plasma vitamin C (∼−0.36 SD in the largest GWAS). As a result, this T allele could be characterized as a genetic proxy of circulating vitamin C and be considered an instrumental variable, given certain assumptions are met [15]. As this variant will be randomly inherited in the population under Mendel’s laws, a causal estimate of the effect of vitamin C can be inferred through the ratio of the association of this allele with an outcome trait of interest (for example, disease risk) relative to its association with circulating vitamin C.

The latest circulating vitamin C GWAS is large relative to many of the previous vitamins (∼52,000 participants) [101]. Eleven genome-wide significant loci were uncovered by this study, with the strongest signal (rs33972313) mapped to *SLC23A1*—1 of the 2 required transporters for vitamin C into target tissues [102]. This *SLC23A1* missense variant has a relatively large effect for a common allele (∼–0.36 SD per T allele), although it has a frequency <8% in all major global populations, and therefore, it is not as common as some of the top signals for other vitamins such as the *RBP4* locus for retinol and the *MTHFR* locus for folate. In vivo ascorbate pharmacokinetic data has suggested that the rs33972313 missense variant reduces the transport capacity of this solute carrier, with a mechanistic explanation for this on circulating abundance of vitamin C ascribed to diminished renal reabsorption [103]. In an earlier GWAS, Timpson et al. [104] estimated that each rs33972313 minor allele was associated with ∼–6 μmol/L reduction in circulating L-ascorbic acid pooled across multiple cohorts. To contextualize this effect size, a normal serum vitamin C measurement can be defined between 40 and 250 μmol/L [105], although the difference between hypovitaminosis C (12–39 μmol/L) and severe deficiency (≤11 μmol/L) is ∼4.5 times that of the estimated effect of a heterozygote rs33972313-T carrier (≤8% of population). The second most significant signal in the largest vitamin C GWAS is plausibly mapped to *SLC23A3*, an orphan receptor with sequence homology to *SLC23A1* but with a less characterized function. Overall, the mean variance explained by all 11 vitamin C-associated variants across 3 cohorts was ∼1.87%. The largest GWAS also reported some evidence of a negative genetic correlation between vitamin C and fasting insulin; however, they found no evidence of a causal effect on liability to type 2 diabetes or on continuous glycemic traits using MR. This null relationship between vitamin C and diabetes accords with previous RCT evidence [106], and further highlights how MR could be used to better triage the necessity of an RCT, thereby saving significant time and resources. Beyond that study, vitamin C-associated variants have been used for MR in several other studies (Figure 3), although no phenome-wide analyses have been performed like some other vitamins. For instance, Fu et al. [107] conducted an MR study that used several cancers as outcome traits that found no strong evidence for an effect on liability to any cancer, although the effect of the functionally relevant *SLC23A1* variant was not specifically tested alone. In another study that investigated a hypothesis-free examination of dietary factors on risk for endometrial cancer, there was some evidence for a risk-increasing effect of circulating vitamin C [108]. While multiple MR models were marginally statistically significant in testing this relationship, this estimate would benefit from analyses that tested the *SLC23A1* by itself as the most biologically interpretable vitamin C IV available.

#### Potential clinical implications and future directions from vitamin C GWAS

The *SLC23A1* locus is one of the largest effect size common variant signals observed for a vitamin; however, there is no evidence currently to support its clinical use with respect to tailoring diet or supplement usage. It would be beneficial to investigate the potential utility of *SLC23A1* genotypes compared with a vitamin C PGS in populations for which vitamin C is of clinical interest, for example, those with chronic wounds. As sample sizes increase, the effect size of a vitamin C PGS should also continue to rise, although as SNP heritability estimates are lacking the theoretical upper bound of this effect size remains unknown. So far, MR has helped to resolve that there is no strong evidence of a relationship between vitamin C concentration and cancer, except for potential endometrial cancer which requires further investigation [108]. In general, genetics has already demonstrated how it could be used to triage RCT opportunities and prevent resource wastage, such as the null effect of vitamin C on liability to type 2 diabetes estimated through MR that was previously seen in RCTs [101]. As with the other vitamins, larger, more diverse samples are needed, particularly to better characterize variation that impacts key aspects of vitamin C physiology beyond transporter proteins. Causal inference using vitamin C IVs would also benefit from future studies that take a hypothesis-free, phenome-wide approach, with the *SLC23A1* missense variant by far the most useful genetic IV at present.

### Vitamin D

Vitamin D, while technically a hormone as it can be endogenously synthesized via a photochemical reaction with ultraviolet-B radiation from sunlight, is still classified as a fat-soluble vitamin, and thus, will still be discussed herein, with extensive review of the dietary sources, biochemistry, and clinical relevance found elsewhere [107,109]. There are 2 main forms of vitamin D—ergocalciferol (vitamin D_2_) and cholecalciferol (vitamin D_3_). Both vitamin D forms produced endogenously and from diet are not active, with 2 metabolic steps to become activated. First, vitamin D_2_ and D_3_ are hepatically hydroxylated to ercalcidiol [25-hydroxyvitamin D (25(OH)D_2_)] and calcifediol [25(OH)D_3_], respectively [[110], [111], [112]]. The second step involves the enzymatic conversion of 25(OH)D_2/3_ to the biologically active form calcitriol [1,25 dihydroxyvitamin D (1,25(OH)_2_D)] [110,111], although strictly speaking calcitriol is specifically the metabolite of calcifediol [1,25(OH)_2_D_3_], while 1,25(OH)_2_D_2_ is sometimes referred to as ercalcitriol [113]. Forthwith, we denote 25(OH)D to represent both the D_2_ and D_3_ forms, with plasma or serum 25(OH)D the most common biomarker of vitamin D status [114]. However, it should be noted that some clinically used 25(OH)D immunoassays are preferentially sensitive to calcifediol [115]. The widespread measurement of 25(OH)D in biobanks and other studies has accelerated progress on GWAS of the concentration of circulating markers of vitamin D status far beyond that of other vitamins, as discussed below.

#### Current findings from vitamin D GWAS

Of all the vitamins, vitamin D has had by far the most comprehensive investigation of its genetic architecture through GWAS, mainly with respect to circulating 25(OH)D. Very large 25(OH)D GWAS have been performed (*N* > 400,000), facilitated by the measurement of 25(OH)D in the first blood biochemistry panel of the UKBB. Two notable UKBB GWAS have been performed that were published at similar times, with the Revez et al. study also meta-analyzing available signals with previous consortium based 25(OH)D GWAS [116,117]. The genetic architecture of vitamin D concentration presents as polygenic; in the Revez et al. [116] study 143 loci were associated with circulating 25(OH)D at genome-wide significance. This study directly estimated the heritability of 25(OH)D using a subset of related UKBB participants at ∼32%, with SNP heritability (directly indexed by GWAS) at ∼13%. The inferred biological significance of these GWAS signals (based on proximal genes) related to factors directly linked to vitamin D biochemistry (for example, *CYP2R1*, *GC*, and *SULT21A*), lipid biology (for example, *PCSK9*, *LIPC*, and *CETP*), and skin-related properties (for example, *FLG*, *DSG1*, and *PADI1*). The signals also refined evidence of novel mechanisms of enzymatic related influences on 25(OH)D biochemistry, such as 2 members from the 17-beta dehydrogenase family (*HSD17B1* and *HSD3B1*). The effect sizes of the lead SNPs on 25(OH)D ranged from very small (∼0.004 SD per allele) to moderately large (∼0.377 SD per allele). Variation in the enzyme encoded by *CYP2R1* represents the largest effect size association with 25(OH)D, with a direct mechanistic link to 25(OH)D as this enzyme catalyzes 25(OH)D synthesis through its 25-hydroxylase activity[118]. In the Manousaki et al. [117] GWAS, this signal was also found to be associated in a small sample with 1,25(OH)_2_D. The second most significant implicated gene is *GC* that encodes the vitamin D binding protein (gc-globulin, aka DBP). To understand the effect size of the *GC* locus, in an earlier study, individuals sampled from the Framingham Heart Study (*N* ∼ 5700) who took vitamin D supplements had a mean 25(OH)D measurement that was ∼9 nmol/L larger than those who did not, an effect size similar to that estimated in that study of each *GC* lead SNP allele (∼10 nmol/L) [119]. There are 2 missense variants that determine key isoforms of the vitamin D binding protein that have marked differences in allele frequency across genetically defined ancestries [120]. The Revez et al. study also conducted the first known investigation of genetic effects on the variance of a vitamin, rather than just mean effects indexed by traditional GWAS [116]. These so-called “variance quantitative trait loci” have been suggested previously to be enriched for nonadditive effects, such as GxE [121,122]. Independent genetic impacts on the variance of circulating 25(OH)D have been identified (*N*_Loci_ = 25), with evidence of interaction of some of these signals with the season of blood draw. Genetic effects on 25(OH)D have also been investigated in genetically defined non-European ancestral populations, and while these studies are lower powered than European counterparts, it represents important progress in this area given these diverse data are unfortunately still lacking for most other vitamins. In the largest such study that leveraged diverse participants from the UKBB, the *GC* signal was also found to influence 25(OH)D across different ancestries, although it did not quite reach genome-wide significance in participants with East Asian genetic ancestry [123]. A recent GWAS of circulating vitamin D binding protein has also been released from samples collected from neonates (*N* ∼ 65,589), as well as an older GWAS from adults (*N* ∼ 1380) [124,125]. The heritability of vitamin D binding protein is much larger than vitamin D (68% in neonates and 60% in adults), indicating a smaller environmental component of the vitamin D transporter concentration. It should be noted that adjustment for the so-called GC diplotype (genotype of 2 missense variants, described above) decreased the vitamin D binding protein heritability estimate to ∼35% of the remaining variance.

**FIGURE 4:**
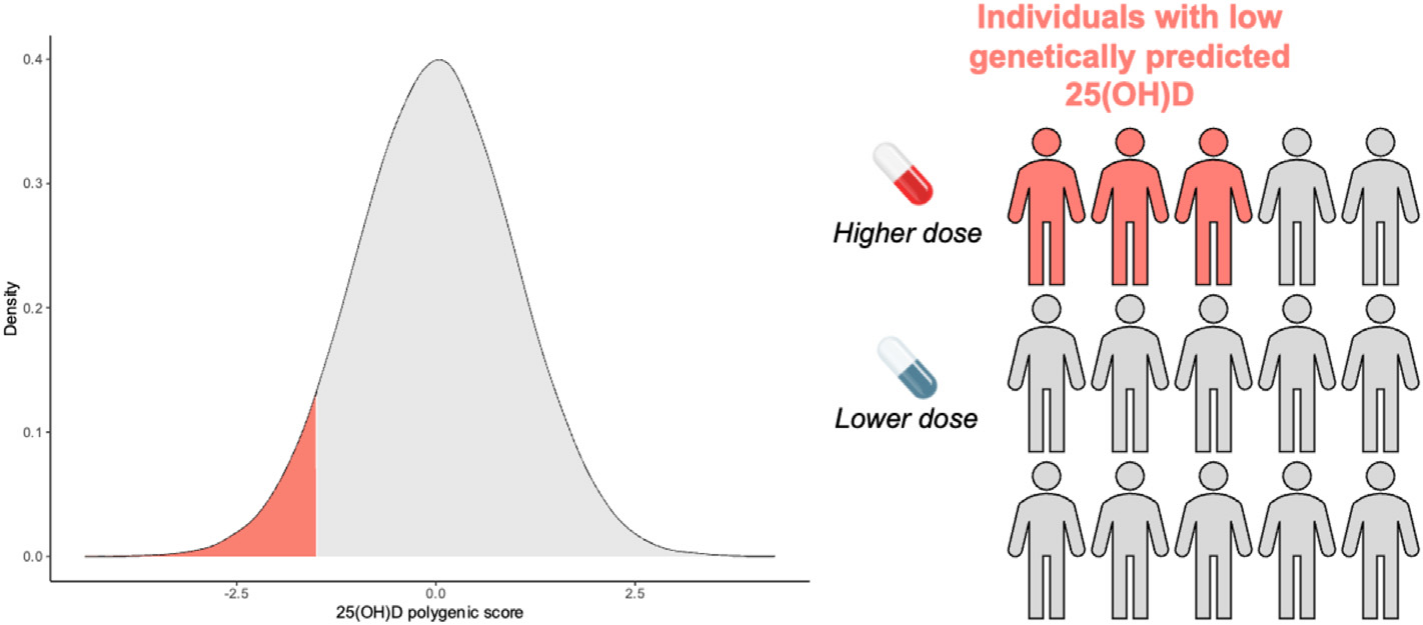
Using polygenic scores to predict circulating 25(OH)D concentrations and its implications of personalized supplementation. The genetic predisposition for higher or lower circulating 25(OH)D can be indexed in an individual using a polygenic score (PGS). Specifically, this summates the alleles carried by an individual weighted by their effect size (association with 25(OH)D) derived from a GWAS conducted in an individual sample. To render these scores interpretable at an individual level, they need to be scaled relative to the distribution (for example, mean and standard deviation) of this score in a comparable population. Individuals who score low after scaling, highlighted on the kernel density estimation plot in this figure using an arbitrary threshold of the lowest 20% of the population, have a lower genetically predicted 25(OH)D than most of the population. This information could be utilized to personalize supplement dosage such that those with low polygenic scores receive higher dosages, as well as receive more pervasive monitoring in clinical situations 25(OH)D is a clinically important sequala, such as malabsorptive conditions.

PGSs of 25(OH)D, which represent a metric of genetically predicted vitamin D, have also been investigated more thoroughly than scores for any other vitamin. In the Revez et al. GWAS, maximal out of sample variance explained by a 25(OH)D PGS was in the region of 10% [116]. A prior study investigated the performance of 25(OH)D PGS in both European and African ancestry participants, albeit with less predictive accuracy in African ancestry participants, highlighting the need for greater diverse samples [126]. The effect size estimated for the PGS when comparing the population extremes in this study is contextualized as a decrease in 25(OH)D (for carriers of a PGS lower than 90% of the population) that would require an additional ∼500 international unit vitamin D intake to maintain equivalence compared with those with PGSs in the top 10% of the population. However, comparing the population extremes is less clinically relevant, and may overinflate the utility of a score. A potentially more informative design to expedite clinical uptake is to examine how a 25(OH)D PGS may influence response to supplementation or other related interventions to vitamin D. In a small Finnish study (*N* = 96), Sallinen et al. [127] used a 2 SNP 25(OH)D score (*GC* and *CYP2R1* signals) to investigate tailoring advice about vitamin D supplement dosage use using genetics, as well as assessing participant attitudes to the use of genetics in this context. This is not a true PGS as it does not weight variants by their effect size, but rather counts the number of total effect alleles (0–2) from both the *GC* and *CYP2R1* variants combined (sometimes referred to as an allelic score). Nonetheless, there was some evidence to suggest that tailoring the dosage of vitamin D supplements considering the number of 25(OH)D-associated alleles was effective in reducing the prevalence of vitamin D deficiency in the study. While this was a small study, it was particularly notable that participants were only advised of supplement dosage, rather than specifically provided supplements, with the study showing that vitamin D supplementation usage increased following digitally delivered genetic counseling and that receiving this genetic information was well tolerated in terms of psychosocial outcomes. This genetic approach to tailoring supplementation may also be useful in specific disease contexts where vitamin D deficiency is a common sequala. For instance, in a small study of newborns diagnosed with cystic fibrosis, in which vitamin deficiencies arise because of malabsorption, there was evidence that a 25(OH)D PGS was associated with time on supplementation to achieve adequate 25(OH)D, although there was no conclusive relationship with overall supplementation nonresponse that would likely need larger samples [128]. Overall, these preliminary data warrant further characterization of the interplay between genetic propensity for 25(OH)D and response to supplement intervention (Figure 4).

Causal inference using genetic variants associated with 25(OH)D has also received considerable attention in the literature, both in terms of the effect of 25(OH)D as an exposure and effects on 25(OH)D as an outcome. Importantly, there have been phenome-wide and hypothesis-free MR studies using 25(OH)D [[129], [130], [131]]. The only strong evidence from these studies was a putative protective effect of vitamin D on liability to the autoimmune disease Multiple Sclerosis (MS) [131]. In the Meng et al. study, the authors also performed a systematic review of previous 25(OH)D MR studies and also highlighted relatively strong evidence that genetically proxied 25(OH)D is protective for MS [129,[132], [133], [134]], a finding further shown using instruments of vitamin D binding protein after *GC* diplotype adjustment, although this is a less biologically specific instrument than *GC* diplotype unadjusted estimates as adjustment removes *GC cis*-acting effects [124]. Despite this relatively strong genetic evidence, and the latitudinal gradient observed for MS risk, clinical trials of vitamin D supplementation in MS have yet to be successful [135]. It is worth noting that sample sizes for these trials have still been relatively small and the number of individuals with clinically evident vitamin D deficiency (25(OH)D < 50 nmol/L) was limited. In future, the genetic adjustment of observed 25(OH)D may prove useful to identify a subset of individuals who would benefit from high-dose vitamin D supplementation in the context of conditions such as MS. Overall, confounding pleiotropy from the now > 100 25(OH)D-associated loci has also proven an issue in causal inference studies of this measure [116], highlighting the importance of specifically considering signals with direct interpretable effects on vitamin D biochemistry. Emerging methods in nonlinear MR have been applied to 25(OH)D, which is likely clinically relevant; however, these have been methodologically challenging to interpret [136]. Finally, there is also somewhat strong evidence that liability to several disorders, such as schizophrenia and bipolar disorder, are causally associated with reduced circulating 25(OH)D [116]. potentially through changes in lifestyle-related behaviors (for example, less exposure to sunlight), among other theorized mechanisms, as reviewed previously [137]. However, the interpretation of effect sizes upon using a binary exposure in MR is predicted on some key statistical challenges [138].

#### Potential clinical implications and future directions from vitamin D GWAS

As the most well-studied vitamin genetically, vitamin D GWAS hold the potential to better refine the need for vitamin D related interventions at the wider population. Indeed, vitamin D supplementation RCT have been performed in very large sample sizes, at significant cost, but with almost universally null findings for individuals who are not deficient [109]. However, it is conceivable that individuals with depleted 25(OH)D PGS may receive an outsized benefit from supplementation, with a genetic index of 25(OH)D capturing longer-term effects on vitamin D biology compared with that of a cross-sectional 25(OH)D measurement. A future direction would be also to consider genetic effects on the variance of 25(OH)D to further explore longitudinal trends. Genetic stratification of existing vitamin D trials where results were unclear or exhibited some significant post hoc findings should be prioritized where viable. Genetics guided approaches in some priority populations may also identify individuals who do not require supplements if they carry a high 25(OH)D genetic burden. Using MR, there is some evidence to suggest the investigation of vitamin D supplementation in MS is warranted[109,132], although early trials have been null. Moreover, in more clinically specific areas such as malabsorptive conditions (for example, cystic fibrosis) where vitamin D deficiency is common, 25(OH)D PGSs hold promise to personalize aspects of supplement indication such as dosage, although more work is needed to investigate efficacy and feasibility of such an approach. We contend that these malabsorptive conditions likely represent the area of medicine where vitamin D genetics offers the most readily apparent path to translation. Further interrogation of vitamin D GWAS also may provide additional insight into pathways such as the impact of lipids on vitamin D physiology that may be clinically relevant. Interestingly, genetic liability to several disorders (for example, schizophrenia) has also been causally linked to 25(OH)D abundance [116,137], which could have implications for monitoring for vitamin D deficiency in particular clinical contexts. Despite the extensive genetic study of vitamin D thus far, at least as compared with other vitamins, there are still several future directions for the field. One such example includes a concerted effort to collect genetic data from cohorts that measure the biologically active form of vitamin D 1,25(OH)_2_D, as well as more RCT of vitamin D supplementation. While there has been some progress to boost ancestral diversity and study nonlinear genetic effects on 25(OH)D, these should be further expanded. Finally, from a clinical implementation perspective more work is needed to characterize whether PGS should be constructed from the largest, most significant signals (for example, *GC*, *CYP2R1*, etc.) or capture the more expansive polygenic signal. Current evidence suggests that a more discrete score which captures genome-wide significant signals only performs optimally for 25(OH)D [116].

### Vitamin E

Vitamin E is a fat-soluble vitamin family comprised of 8 vitamers: alpha-, beta-, gamma- and delta-tocopherols and tocotrienols, as discussed in detail previously [139,140]. Bioavailability varies between these vitamers, with alpha-tocopherol being by far the most predominant form found in tissues arising from its increased bioavailability [141]. Serum or plasma concentrations of alpha-tocopherol are usually preferred biomarker of vitamin E status, however, erythrocyte lysis susceptibility when challenged with hydrogen peroxide has been suggested as a functional biomarker of vitamin E status [142]. Given the salience of the alpha-tocopherol vitamer, genetic studies of vitamin E have focused on circulating alpha-tocopherol measurements.

#### Current findings from vitamin E GWAS

The largest dedicated GWAS of circulating alpha-tocopherol was drawn from ∼5000 participants and published in 2011 [143]. Three signals reached genome-wide significance in this study, with the strongest signal on chromosome 11 (rs964184 lead SNP). This chromosome 11 locus has a strong association with lipids, containing genes including *APOC* and *APOA4*, and while this GWAS did adjust for total cholesterol, the direct effect on alpha-tocopherol independent of lipid abundance is yet to be thoroughly characterized. The second most significant signal is a missense variant in the gene *CYP4F2* (rs2108622 and V433M), which has a direct mechanistic relationship with alpha-tocopherol and is less likely to be directly confounded by genetic influences on lipoproteins. Specifically, tocopherols are metabolized via *CYP4F2-*catalyzed ω-hydroxylation into water-soluble compounds that can be excreted [144]. Functional interrogation of this missense locus suggests that it lowers the specific activity of *CYPF42* toward alpha-tocopherol, and thus, boosts serum concentrations [145]. This *CYPF42* signal also has implications for vitamin K biochemistry and warfarin dosage that will be discussed in a subsequent section of this review. When considering all 3 lead SNPs identified, the variance explained in alpha-tocopherol by these signals was ∼1.7%, a comparable proportion to genome-wide significant signals for other vitamins but this requires larger samples for confirmation. A follow-up study by the same group [146], investigated genome-wide influences on serologic response to long-term vitamin E supplementation and found the same lipid associated chromosome 11 signal at genome-wide significance, while the *CYP4F2* signal was just above this threshold. At the time of writing, the largest sample size GWAS of alpha-tocopherol was a recent high-throughput metabolomics GWAS (*N* ∼ 20,000) that replicated the chromosome 11 finding but surprisingly did not replicate the *CYP4F2* finding at genome-wide significance [147]. Larger sample sizes are still needed to fully appreciate genome-wide heritability and genetic architecture of alpha-tocopherol and other vitamers. In terms of PGSs, a recent CSF metabolomics study used a genome-wide Ridge regression weighted score of alpha-tocopherol that explained ∼2.7% of the phenotypic variance but estimations of predictive capability in serum measures are still lacking [148]. There has also been some work applying findings from vitamin E GWAS for causal inference, although hypothesis-free studies have not been performed and currently available genetic instruments are difficult to biologically interpret as even *CYP4F2* is associated with biochemistry for other vitamins and metabolites. MR applied across 10 common cancers revealed some evidence of a risk-increasing effect of vitamin E on bladder cancer but a protective effect on breast cancer; however, the breast cancer outcome loses statistical significance in multivariable models that genetically control for lipid related factors [149]. We caution that given the small sample size of vitamin E GWAS used in that study, a lack of conditional instrument strength may be a concern in these multivariable MR models and larger datasets are needed to better apply genetically informed causal inference of vitamin E vitamers [150].

#### Potential clinical implications and future directions from vitamin E GWAS

The utility of GWAS of vitamin E status related markers is still somewhat hampered by small sample sizes and some uncertainty related to how to best adjust for potential confounding lipid effects in the genetic context. However, the signal mapped to *CYP4F2*, has previously received some clinical consideration. For instance, Xu et al. [151] tested the moderating effect of the *CYP4F2* rs2108622 on longitudinal changes in vitamin E after supplementation, nominally replicating previous evidence for an association of *CYP4F2* with response to vitamin E supplementation, described above [146]. In that same study, there was some evidence to suggest that longitudinal changes in vitamin E after supplementation are associated with a reduction in age-related lung function decline, whereas analysis of vitamin E treatment as a categorial variable was not [151,152]. While this particular indication (lung function) requires further consideration in terms of the utility of vitamin E supplementation, it does reinforce that genetic influences on vitamin status biomarkers may be useful to refine any potential utility of supplementation for chronic disease, whereby trial/cohort analyses can be conditioned on underlying genetic predisposition for factors such as metabolism. Analogous to the discussion of previous vitamins, a more expansive vitamin E PGS also warrants more investigation, but larger GWAS sample sizes are needed. The confounding influence of lipids on serum alpha-tocopherol could be mitigated through genetic adjustment for lipids, such that collider biases that do not arise when genetic studies covary for directly measured heritable covariates, as discussed previously [153]. MR may also be useful to better triage clinical indications for which supplementation or dietary changes may, or may not, be efficacious, although at present there are only few IVs for this vitamin which lack biological specificity. Specifically, even the most interpretable genetic signal for vitamin E (*CYP4F2*) is pleiotropically associated with vitamins and other biochemical traits, as discussed in the proceeding section.

### Vitamin K

Vitamin K denotes a family of fat-soluble vitamers which cannot be produced endogenously by humans, and therefore, must be obtained from the diet [154]. There are two central vitamers, phylloquinone (vitamin K_1_) and menaquinones (vitamin K_2_), which differ in their chemical structures with different side chains[155]. Vitamin K_1_ food sources are primarily plant-based foods such as green leafy vegetables and vegetable oils, whereas vitamin K_2_ sources are animal based including meat, eggs and dairy [154,156]. A detailed review of the dietary sources, physiology, and implications for human health and disease can be found elsewhere [157]. The primary two biomarkers used include blood phylloquinone, which is reflective of dietary intake, but its short half-life means that it is suggested to reflect recent intake. The other proposed biomarkers are prothrombin time and partial thromboplastin time, which is inversely associated with higher thrombin time being reflective of vitamin K deficiency. However, consensus guidelines do not consider either as sensitive biomarkers of vitamin K status [158].

#### Current findings from GWAS vitamin K GWAS

Progress in unraveling the genetic architecture of vitamin K status has been hindered by the lack of consensus on suitable biomarkers, as described above. Relatively large GWAS of prothrombin time have been performed, with the largest such GWAS from a Japanese cohort (*N* ∼ 58,000) [159]. Unsurprisingly, the strongest signals for this phenotype reflect direct genetic effects on prothrombin genes, and thus, the relevance of genetic studies of prothrombin time directly on vitamin K biochemistry is not clear without additional data. Despite its limitations as a biomarker, there has been one dedicated GWAS of circulating phylloquinone, albeit in a small sample size (*N* ∼ 2138) [160]. No genome-wide significant loci were uncovered. However, the alpha-tocopherol associated *CYP4F2* missense variant was suggestively significant (*P* < 1 × 10^–6^) in the same direction whereby the *T* allele boosts serum phylloquinone. This enzyme also has a direct mechanistic link to vitamin K biology, with in vitro data revealing that the missense variant reduces *CYP4F2* catalyzed phylloquinone side chain oxidation [161]. This *CYP4F2* was known to be clinically relevant in previous work related to warfarin dosage and is classified as a “very important” pharmacogene according to PharmGKB [162], and has also been found in GWAS of warfarin dosage [163]. Individuals who carry the carriers of the CYP4F2 V433M require larger doses on warfarin, likely because of elevated phylloquinone that influences coagulation and clotting. In future, the effect of this *CYP4F2* signal should be investigated in larger samples with more informative vitamin K biomarkers if greater consensus can be reached. The application of causal inference using vitamin K genetic signals as instruments is also made difficult by the limitations of using circulating phylloquinone and low statistical power. Even the *CYP4F2* signal, which is directly linked to vitamin K metabolism, displays pleiotropic associations with other factors such as vitamin E. An MR study of circulating phylloquinone on liability to type 2 diabetes did find some evidence for a protective effect that was relatively consistent across 4 suggestively associated alleles from the Dashti et al. GWAS [164], and while there have been some other MR studies [165,166], all of these are difficult to interpret because of the limitations described above.

#### Potential clinical implications and future directions from vitamin K GWAS

Without more data to refine the specificity of vitamin K status biomarkers, the clinical utility of vitamin K-related GWAS is somewhat limited, particularly as sample sizes remain very small. The circulating phylloquinone GWAS described above, however, does consolidate the clinical applicability of *CYP4F2* as a pharmacogene for anticoagulants given its interrelationship with vitamin K physiology. A key question yet to be explored is if there are any dietary implications of phylloquinone-associated *CYP4F2* variation. For example, investigating the interplay between *CYP4F2*, warfarin dosage, and intake of foods rich in vitamin K. This could allow for more specific advice regarding vitamin K intake recommendations, rather than just a general rule to avoid vitamin K rich foods, such as leafy greens. The causal inference work discussed above has triangulated with some previous clinical data; however, IVs are weak because of the lack of a gold-standard biomarker. Future work that characterizes genetic influences on vitamin K_2_ in sufficiently large samples would also aid efforts in this area.

## Conclusions and future directions

In this review, we outline the progress in the field to date in using hypothesis-free GWAS to explore the genetic architecture of biomarkers of vitamin status. We assert that the key utility of these data presents in terms of *1*) characterizing genetic regulatory influences on vitamin biochemistry (Table 1) [28,29,38,43,62,83,101,117,124,143,160,184] *2*) better understanding the relationship between vitamins and human health, and *3*) development of PGS that index individual genetic burden of vitamin biomarker associated variation. Individual signals and PGS can also be used for GxE analyses as sample sizes increase and statistical methodology further innovates. Maximizing the utility of vitamin GWAS for discovery-based research and clinical translation will require a concerted effort to overcome the limitations inherent to each vitamin. Overall, vitamin D represents by far the most well-characterized vitamin genetically, with vitamin A, vitamin C, vitamin B_9_, and vitamin B_12_ also well powered to discover genome-wide significant loci but still somewhat underpowered for more fully studying their overall genetic architecture. Progress with respect to several of the B vitamins, as well as vitamin K, is hampered by a lack of either informative biomarkers that are difficult to measure at scale or a consensus about suitable biomarkers[2,71]. Below, we provide some recommendations for future work that are broadly applicable across vitamins for which GWAS are feasible to apply.

1. Collection and analysis of vitamin GWAS from diverse genetically defined ancestral groups.

**TABLE 1:** Key findings from vitamin status GWAS.

Vitamin/vitamer^1^	Published variance explained by genome-wide significant signals	Published SNP heritability estimates^2^	Key genes with direct impact on vitamin biochemistry	References
Retinol	2.3% (*RBP4* and *TTR* only)^4^	7%–15%	*RBP4* (transport) *TTR* (transport)	Mondul et al. [29], Reay et al. [28]
Beta-carotene	1.9%^5^	–	*BCO1* (metabolism)	Ferrucci et al. [39]
Vitamin B_3_ (1-MN and 2-PYR)^3^	–	–	*ACMSD* (synthesis)	Yin et al. [43]
Vitamin B_6_	–	–	*ALPL* (metabolism)	Tanaka et al. [62]
Vitamin B_9_	1%^4^	–	*MTHFR* (metabolism) *FOLR3* (receptor)	Grarup et al. [83]
Vitamin B_12_	6.3%^4^	–	*CD320* (receptor) *TCN2* (transport) *ABCD4 (*transport) *MMAA* (coenzyme) *MMACHC* (intracellular transport) *TCN1* (transport) *CUBN* (absorption) *MMUT* (coenzyme)	Grarup et al. [83]
Vitamin C	1.87%^4^	–	*SLC23A1* (transporter)	Zheng et al. [101]
Vitamin D [25(OH)D]	3.6%–7.5%^5^	13%	*GC* (transport) *CYP2R1* (synthesis) *CYP24A1* (metabolism) *SULT21A* (metabolism)	Ahn et al. [167], Revez et al. [124], Manousaki et al. [117]
Vitamin E (alpha-tocopherol)	1.7%^4^	–	*CYP4F2* (metabolism)	Major et al. [143]
Vitamin K (phylloquinone)	–	–	*CYP4F2* (metabolism)^6^	Dashti et al. [160]

1 Vitamin B_1_, Vitamin B_2_, Vitamin B_5_, and Vitamin B_7_ not listed because of either insufficient consensus of suitable status biomarkers or lack of GWAS on said biomarkers.

2 Heritability explained by signal captured by GWAS.

3 Denotes major niacin metabolites: 1-methylnicotinamide (1-MN) and 1-methyl-2-pyridone-5-carboxamide (2-PYR).

4 Within sample estimate.

5 Independent sample from discovery GWAS.

6 The association between *CYP4F2* and circulating phylloquinone was only suggestively significant (*P* < 1 × 10^–6^).

A major limitation of most genetic studies of vitamin status markers is that they have only been performed in genetically defined European ancestry cohorts. This not only hinders discovery power and genetic fine-mapping [168], but also could result in inequitable translation of genetic findings—for example, PGSs that perform worse in non-European genetically defined ancestral groups [169]. In the interim, statistical methodologies to improve the “portability” of PGS across ancestries may assist in mitigating these challenges to some extent. However, it should be noted that for other quantitative traits there has been heterogeneity in the magnitude of reduction of PGS performance in non-European cohorts across different genetically defined ancestral groups. For instance, PGS of blood pressure, European-trained PGS performed similarly in some other genetically defined ancestral groups but not others [170,171]. Methodological advances to further address issues in cross-ancestry PGS also continue to emerge and will be useful in this context [172].

2. Careful selection of instruments for causal inference using genetic proxies of vitamin status markers that balances biological specificity with statistical power.

The use of genetically informed causal inference approaches (for example, MR) holds great potential to further refine our understanding of how vitamins influence human health, particularly given the concerns about unnecessary overuse of supplements in the general population. There remain some barriers to fully realizing this potential, specifically related to the selection of so-called “genetic proxies” of vitamin status. One strategy for this is to select only variants with a direct, unambiguous link to the vitamin phenotype of interest—for example, *RBP4* and retinol, and *SLC23A1* and vitamin C. Alternatively, there exists a suite of MR methods that can leverage many trait-associated variants as IVs with less strict assumptions, and therefore, all genome-wide significant signals could be used and power increased [16]. For instance, hundreds of genome-wide significant loci have been used in published MR models of 25(OH)D [116]. While the inclusion of many variants as IVs facilitates the testing of several different assumptions and modeling approaches, it comes at the cost of biological specificity. We recommend that both of these approaches should be deployed together and compared, where possible, as well as modeling genome-wide genetic correlations if there is power to do so [[173], [174], [175]]. Similar considerations have been discussed using specific protein abundances as instruments for MR. For instance, Zheng et al. [176] in a landmark MR study of thousands of proteins across the human phenome contend that *trans* acting instruments (similar to genetic variants without a direct impact on vitamin biochemistry in this context) are prone to pleiotropy but could increase statistical power to examine downstream effects, as well as testing metrics such as heterogeneity between instruments. Lastly, we recommend triangulating MR with other approaches when inferring causality where feasible to do so [177].

3. Pairing GWAS of vitamin status biomarkers with well-powered GWAS of key vitamin receptors, transporters, and metabolic enzymes.

With the increasing advent of large GWAS of proteomic traits, such as that performed in cohorts such as the UKBB and deCODE [178,179], *cis* and *trans* acting genetic influences on the expression of key components of vitamin biochemistry can also be studied—for example, transporters, receptors, and key enzymes involved in metabolism. These data allow for genetic estimates on vitamin status biomarkers to be triangulated with how implicated genes/pathways also impact machinery directly related to its physiology. The utility of this approach has already been shown with respect to GWAS vitamin D binding protein and 25(OH)D, where *trans* acting SNP effect sizes on vitamin D binding protein could be examined for their association with 25(OH)D [124]. MR using instruments for specific components of vitamin physiology may also provide more scope for sensitivity analyses related to testing heterogeneity and investigation of tissue-specific effects.

4. Using genetics and high-throughput metabolomics to characterize novel molecules related to vitamin status to increase discovery power.

High-throughput metabolomic profiling of large, biobank scale cohorts which are also genotyped has accelerated GWAS discovery for some vitamins. These datasets also provide an opportunity to characterize novel molecules related to vitamin status. Specifically, genetic correlation and causal inference approaches between known vitamin status biomarkers and uncharacterized metabolites may reveal new avenues for genetic investigation to unravel the genetic architecture of vitamin physiology. These kinds of untargeted metabolomics approaches are already gaining popularity with respect to investigating dietary patterns [180].

5. Further exploration of nonlinear effects, including GxE interactions.

There has been some preliminary work to apply best practice approaches in modern statistical genetics to detect nonadditive genetic effects on vitamin status biomarkers, although well-powered sample sizes for such work are only available for 25(OH)D. We recommend that the most tractable approach to detect nonadditive joint effects, such as gene-by-diet interactions, is to first screen for genetic impacts on phenotypic variance, which are enriched for nonadditive signals [121,122], and then probe these signals further. These interaction models must be carefully constructed to avoid issues, such as, excessive heteroskedasticity of residuals and interaction effects with covariate terms [97,181].

6. Studying the clinical applicability of vitamin PGSs in the context of diseases or dietary patterns where vitamin deficiency is common.

Vitamin status biomarker PGS provide an index of genetically predicted biomarker abundance that may be useful to understand individuals that are predisposed to deficiency or toxicity from vitamins. While more work is needed to refine and increase the predictive utility of these scores, we assert that one area where these PGS should receive further study is with respect to populations, dietary patterns or clinical indications where deficiency is common and requires direct clinical management.

7. Further consideration of genetic influences on vitamin status biomarkers that arise because of impacts on intake (for example, taste) and behavioral patterns (for example, exercise, smoking etc.).

In this review, we have focused mostly on genetic effects on biological markers of vitamin status that can be directly linked to their biochemistry. These more readily interpretable effects are important; however, they are an incomplete picture of the genetic architecture of these traits. As sample sizes increase and the polygenic architecture of vitamin GWAS can be more accurately estimated (like has been done for 25(OH)D), we recommend that efforts should also be made to disentangle genetic effects that may arise because of their relationship with factors related to dietary intake, such as taste perception, as well as other behavioral patterns (for example, smoking, physical activity, sleep patterns etc.).

8. Inclusion of researchers and clinicians in vitamin GWAS from diverse specialties that are relevant to these studies—for example, biochemistry, statistical genetics, functional genomics, and dietetics.

Finally, we believe that it is critical to break down silos between disciplines with respect to genetic studies of vitamin markers of vitamin status, although this same principle extends beyond nutrition genetics. Besides the key role of statistical geneticists in performing GWAS, and subsequently leveraging GWAS data, clinical (for example, dietitians) involvement is important to align priorities for study and develop ways to translate findings from these studies. Moreover, the complex and heterogeneous nature of measuring traits that are reflective of vitamin status, and selecting these for GWAS, will benefit from increased collaboration with biochemists and related experts. Biological interpretation of GWAS findings often requires integration with other form of “omics” (for example, epigenetics, transcriptomics etc.). As technological advances in functional genomics continue to rapidly development, experts in the application of these methods are useful to prioritize both in silico and experimental methodology to interpret GWAS loci associated with vitamin phenotypes. Finally, it is also crucial to capture consumer attitudes on the use of genetics in contexts such as clinical dietetics, as well as other potential end-users such as health policy makers.

There are also some overall limitations of GWAS that should also be considered in the interpretation of this study, as reviewed elsewhere [17,182,183]. In particular, it should be emphasized that GWAS signals are associations, and further work is often needed to refine variants causally linked to phenotypes as opposed to those driven by LD [184]. Moreover, GWAS loci must be replicable and careful attention paid to the use of covariates, particularly when those covariates themselves are heritable phenotypes [153]. In conclusion, we believe that a concerted effort is needed to maximize the utility of GWAS of vitamin status biomarkers to fully realize their potential with respect to obtaining greater biological insights and clinically informative findings. The most efficient mechanism to achieve these goals will be an interdisciplinary approach that leverages the diverse expertise of researchers interested in this field and the rapidly evolving landscape of genetic data available for study.

## Author contributions

The authors’ responsibilities were as follows – WRR: design. WRR, EDC, CA, LDH: writing, and all authors have read and approved the manuscript.

## Conflict of interest

The authors declare no conflict of interest.
